# Progress of Crude Oil Gasification Technology Assisted by Microorganisms in Reservoirs

**DOI:** 10.3390/microorganisms12040702

**Published:** 2024-03-29

**Authors:** Shumin Ni, Weifeng Lv, Zemin Ji, Kai Wang, Yuhao Mei, Yushu Li

**Affiliations:** 1 University of Chinese Academy of Sciences, Beijing 100049, China; nishumin22@mails.ucas.ac.cn (S.N.); wangkaiz@petrochina.com.cn (K.W.); meiyuhao22@mails.ucas.ac.cn (Y.M.); liyushu22@mails.ucas.ac.cn (Y.L.); 2 Institute of Porous Flow & Fluid Mechanics, Chinese Academy of Sciences, Langfang 065007, China; jizemin@petrochina.com.cn; 3 State Key Laboratory of Enhanced Oil Recovery, Research Institute of Petroleum Exploration and Development, CNPC, Beijing 100083, China

**Keywords:** crude oil gasification pathway, microbial communities involved in crude oil gasification, factors affecting methanogenic efficiency

## Abstract

Crude oil gasification bacteria, including fermenting bacteria, hydrocarbon-oxidizing bacteria, reducing bacteria, and methanogenic bacteria, participate in multi-step reactions involving initial activation, intermediate metabolism, and the methanogenesis of crude oil hydrocarbons. These bacteria degrade crude oil into smaller molecules such as hydrogen, carbon dioxide, acetic acid, and formic acid. Ultimately, they convert it into methane, which can be utilized or stored as a strategic resource. However, the current challenges in crude oil gasification include long production cycles and low efficiency. This paper provides a summary of the microbial flora involved in crude oil gasification, the gasification metabolism pathways within reservoirs, and other relevant information. It specifically focuses on analyzing the factors that affect the efficiency of crude oil gasification metabolism and proposes suggestions for improving this efficiency. These studies deepen our understanding of the potential of reservoir ecosystems and provide valuable insights for future reservoir development and management.

## 1. Introduction

**Figure 1:**
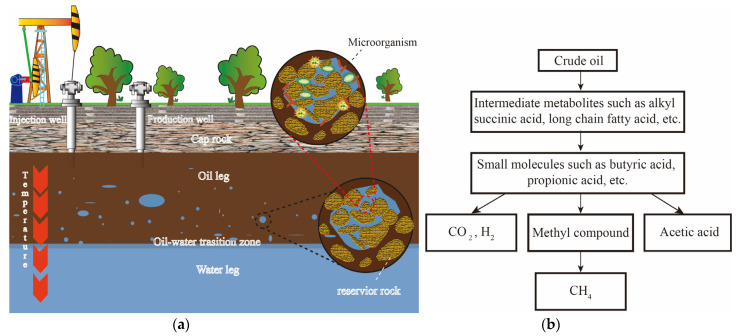
(**a**) Reservoir environment diagram; (**b**) Diagram of the main process of crude oil gasification.

After experiencing natural energy, hydraulic, and chemical displacement, at least 50% of the crude oil in the reservoir cannot be recovered [1], resulting in a decline in the utilization of oil resources. Since the 1980s and 1990s, researchers such as Zengler [2], Anderson [3], and Cheng [4] have confirmed that the synergistic effect of methanogens and methanotrophs can lead to the degradation and conversion of crude oil into methane, thereby reducing resource loss. Consequently, scientists have proposed “bio-gasification” technology for crude oil [5], which aims to degrade the challenging-to-mine hydrocarbons in the reservoir into small molecular substances such as hydrogen and carbon dioxide through microbial action, ultimately converting them into extractable methane [6], as shown in Figure 1.

During the biodegradation of crude oil, hydrocarbon substrates are initially activated through pathways involving fumarate addition, hydroxylation, carboxylation, and methylation [7]. Subsequently, these compounds undergo further degradation into intermediate lower-molecular-weight products, including formate, acetate, hydrogen, and carbon dioxide, via pathways involving beta-oxidation and central metabolism. These processes are primarily conducted by hydrocarbon-oxidizing bacteria, fermenting bacteria, and various reducing bacteria, predominantly within the deferribacteres, firmicutes, and proteobacteria phyla [8,9]. Subsequently, the intermediate products formate, acetate, hydrogen, and carbon dioxide are transformed into methane through pathways that include methylotrophic, acetotrophic, and hydrogenotrophic processes [10,11], which involve various methanogenic archaea. The process of crude oil biodegradation engages a diverse range of microorganisms and enzymes that catalyze intricate metabolic reactions using their own synthetases, facilitating adaptation to extreme environments [12]. Consequently, crude oil biodegradation represents a comprehensive and intricate biochemical process, which, to a certain extent, constrains the efficiency of reservoir crude oil gasification due to various factors. To maximize the efficiency of crude oil gasification, Lin et al. [13] systematically researched the impact of temperature, pH, carbon sources, and other factors, elucidating the straightforward correlations between individual variables and the efficiency of crude oil gasification. As artificial intelligence has advanced, machine learning has demonstrated distinct advantages in fitting predictions and evaluating the significance of multiple variables [14,15]. Building on prior research regarding the correlation between individual variables and the efficiency of crude oil gasification, the integration of machine-learning algorithms is anticipated to enable the evaluation of the significance of multiple variables in the crude oil gasification process [16], thus aiding in the precise control of crude oil gasification in engineering applications.

Microorganisms act on the crude oil in the reservoir, leading to its vaporization and subsequent enhancement of the oil’s utilization rate. Additionally, this process yields clean energy, notably methane, thereby advancing the traditional fossil energy sector towards low-carbon practices. This method is anticipated to emerge as a novel solution for global energy requirements in the future [17,18,19]. Despite the significant potential of crude oil gasification, most research on crude oil gasification remains at the laboratory stage, and the widespread implementation of reservoir crude oil gasification technology has yet to be achieved. While existing studies have outlined the primary reaction pathways of the crude oil gasification process, the assessment of the importance and precise regulation of influencing factors has not been achieved due to the gasification efficiency being influenced by various complex factors, including environmental and substrate factors. This paper extensively examined the current state of reservoir crude oil gasification, with a focus on elucidating the reservoir crude oil degradation flora and metabolic pathways. Furthermore, it summarized, analyzed, and evaluated the factors influencing the efficiency of the metabolic process. Based on this analysis, a series of feasible suggestions were proposed to enhance the efficiency of crude oil gasification, and the substantial potential of reservoir crude oil gasification, facilitated by microorganisms, in terms of increased production and carbon reduction, was identified.

## 2. Microbial Flora Involved in Gasified Degradation of Crude Oil and Their Synergistic Effects

**Figure 2:**
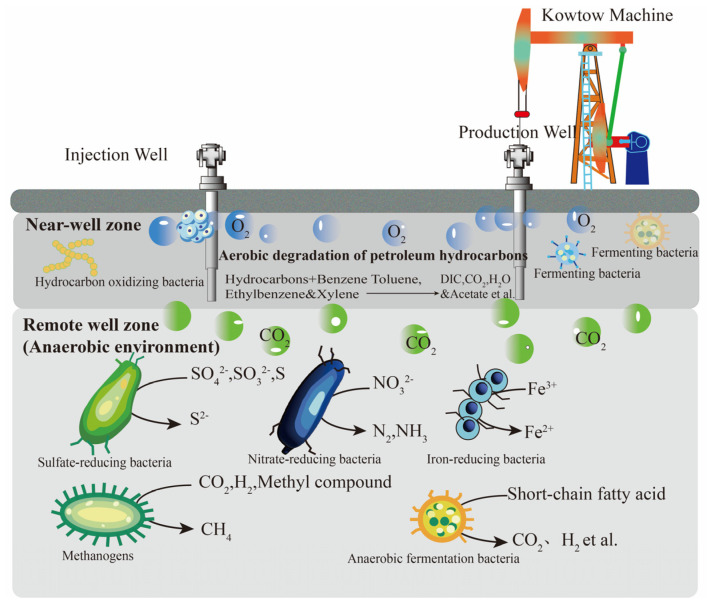
Longitudinal distribution diagram of crude oil degrading bacteria in the reservoir.

The flora involved in crude oil gasification comprises fermentation bacteria, hydrocarbon-oxidizing bacteria, sulfate-reducing bacteria, nitrate-reducing bacteria, iron-reducing bacteria, and methanogenic archaea [14], as listed in Table 1. As the depth increases, the distribution of crude oil gasification bacteria in the oil reservoir exhibits significant vertical variation, as shown in Figure 2. Aerobic bacteria are primarily distributed in the near-well area, while anaerobic bacteria are mainly found in the far-well end. This distribution pattern arises from the inevitable entry of a small amount of oxygen into the reservoir after a series of oil-displacement methods, creating a relative aerobic zone where aerobic fermentation bacteria and hydrocarbon-oxidizing bacteria are activated to oxidize and degrade hydrocarbons. As hydrocarbon-oxidizing bacteria consume dissolved oxygen in water, an anaerobic environment gradually forms, facilitating the activation of anaerobic and facultative aerobic fermentation bacteria for hydrocarbon degradation. Fermentation bacteria, in conjunction with various reducing bacteria, are responsible for converting hydrocarbons into secondary metabolites such as volatile fatty acids, methyl compounds, acetic acid, carbon dioxide, and hydrogen. Ultimately, methanogenic archaea convert secondary metabolites like methyl compounds, acetic acid, and carbon dioxide into the final product, CH_4_. Consequently, the crude oil gasification flora can be broadly categorized into two groups: interacting hydrocarbon-degrading flora and methanogenic bacteria, which are responsible for the initial degradation and final methanogenic processes, respectively.

Researchers isolated, screened, and enriched interbacterial communities involved in crude oil hydrocarbon degradation from diverse habitats [36,37,38,39,40], as shown in Table 2. These communities primarily consist of hydrocarbon-oxidizing bacteria, fermentation bacteria, and various reducing bacteria. Hydrocarbon-oxidizing bacteria thrive in shallow aerobic environments, utilizing hydrocarbons as a carbon source and oxygen as an electron acceptor to fuel their growth. Fermentation bacteria break down macromolecules (e.g., long-chain saturated hydrocarbons and aromatic hydrocarbons) in the reservoir, producing small molecules such as short-chain fatty acids, alcohols, methyl groups, and carbon dioxide in both aerobic and anaerobic settings. Reducing bacteria derive energy from electron acceptors (e.g., NO_3_^−^, Fe^3+^, Mn^2+^, SO_4_^2−^) present in the reservoir’s in situ environment, metabolizing small molecules like nitrogen, carbon dioxide, and hydrogen sulfide in the process. The strains were identified through high-throughput sequencing, 16S rRNA analysis, metagenomics, and transcriptomic techniques. The analysis revealed that the majority of hydrocarbon-oxidizing bacteria (HOB) belong to genera such as *Pseudomonas*, *Staphylococcus*, *Streptobacillus*, *Acinetobacter*, and *Rhodococcus* [41]. These genera have been recognized for their significant role in petroleum hydrocarbon degradation. The isolated fermentation bacteria primarily belong to the genera *Thermococcus, Bacteroidetes*, *Acinetobacter*, and *Haloanaerobium*, among others [15,16,17]. The majority of reduced strains are classified within the genera *Smithella*, *Desulfovibrio*, and *Desulfobulbus.* As biological technology advances, researchers are continuously uncovering new strains. They explore novel metabolic pathways through enriching isolated strains, constructing complex microbial communities and utilizing key metabolic markers and isotope tracers.

Methanogenic bacteria are widely found in extreme anaerobic environments such as deep oil reservoirs [48], activated sludge, marine sediments, etc., where they are responsible for converting acetic acid, methyl compounds, carbon dioxide, etc., produced by the initial degradation process into CH_4_. Methanogenic archaea in oil reservoirs are diverse and can be primarily classified into acetoclastic, hydrogenotrophic, and methylotrophic groups based on their nutritional substrates, as listed in Table 3. In addition, Mayumi et al. [54] confirmed that *Methermicoccus shengliensis ZC-1* isolated from the Shengli oilfield is capable of degrading methoxylated compounds to produce methane, while Cheng et al. [4] demonstrated that the methanogen Ca. *Methanoliparum* can directly degrade alkanes to produce methane. The temperature conditions in anaerobic environments can influence the metabolic pathways of methanogenic archaea and the distribution of dominant genera. Psychrophilic methanogens tend to favor acetoclastic metabolism, and the H_2_/CO_2_ reduction pathway is no longer present at 6 °C. Mesophilic methanogens mostly have both hydrogenotrophic and acetoclastic metabolic pathways and adjust their dominant pathways according to different environmental conditions. Thermophilic methanogens primarily utilize acetate oxidation and hydrogenotrophic methane-production pathways, and most of them are unable to metabolize formate esters, methanol, or trimethylamine for methylotrophic methane production. Hyperthermophilic methanogens tend to favor the hydrogenotrophic metabolic pathway. Notably, Wang et al. [55] found that the main methane producers in high-temperature hot springs, the order archaeoglobales, have both hydrogenotrophic and methylotrophic pathways for methane production, and they can metabolize methanol and produce methane via the hydrogenotrophic pathway at low temperatures (65 °C), while they use the methylotrophic metabolic pathway at high temperatures (75 °C). Furthermore, there are certain species differences in methane production among different types of culture, with acetoclastic methanogens mostly belonging to the *Methanosaeta* and *Methanococcus* genera, while methylotrophic methanogens are mostly classified into the orders Methanococcales, Methanocellales, and Methanomicrobiales.

**Figure 3:**
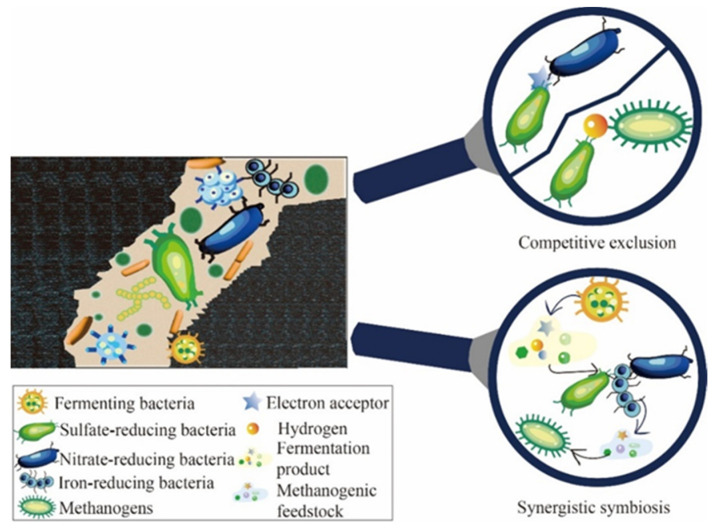
Microbial interaction during crude oil gasification.

Microbial ecological communities in reservoir crude oil gasification exhibit competitive exclusion, mutualistic symbiosis, partial symbiosis, and parasitism among themselves, as depicted in Figure 3. During crude oil gasification, methanogenic archaea and mutualistic hydrocarbon-degrading bacteria engage in a synergistic symbiotic relationship. Specifically, fermenting and methanogenic bacteria within mutualistic hydrocarbon-degrading communities are often closely associated, facilitating electron exchanges through proton flow, formate, or inorganic conductive particles in nanowires [66] and biofilm matrices [67]. Intercalated hydrocarbon-degrading communities break down complex organic compounds like long-chain alkanes and aromatic hydrocarbons into simple molecules such as carbon dioxide, acetate, and formate, providing the necessary carbon source for the growth and metabolism of methanogens [68,69]. Simultaneously, the energy released from methanogen metabolism supports the growth of intercalated hydrocarbon-degrading bacteria. Additionally, sulfate-reducing bacteria, nitrate-reducing bacteria, and hydrogenotrophic methanogens compete for nutrients because of the shared substrate. Due to the higher energy release in the nitrate reduction reaction compared to the sulfate reduction reaction, nitrate-reducing bacteria exhibit greater substrate competitiveness than sulfate-reducing bacteria when utilizing identical substrates. This enables them to competitively inhibit the production of hydrogen sulfide [70,71,72]. Sulfate-reducing bacteria and hydrogenotrophic methanogens can both utilize hydrogen as an electron donor. In sulfate-rich reservoirs, sulfate-reducing bacteria compete with methanogens for hydrogen as a substrate. Hence, to optimize the gasification efficiency of crude oil, it is imperative to judiciously leverage the interaction dynamics among gasification bacteria. This involves maintaining a balanced ratio of nitrate-reducing bacteria to sulfate-reducing bacteria, as well as sulfate-reducing bacteria to hydrogen-trophic methanogens. Such equilibrium ensures the timely and effective decomposition of intermediate metabolites. Additionally, introducing a suitable quantity of hydrogen-producing and acetogenic bacteria is essential to maximize the efficacy of methanogens.

## 3. Metabolic Mechanisms of Reservoir Crude Oil Gasification

**Figure 4:**
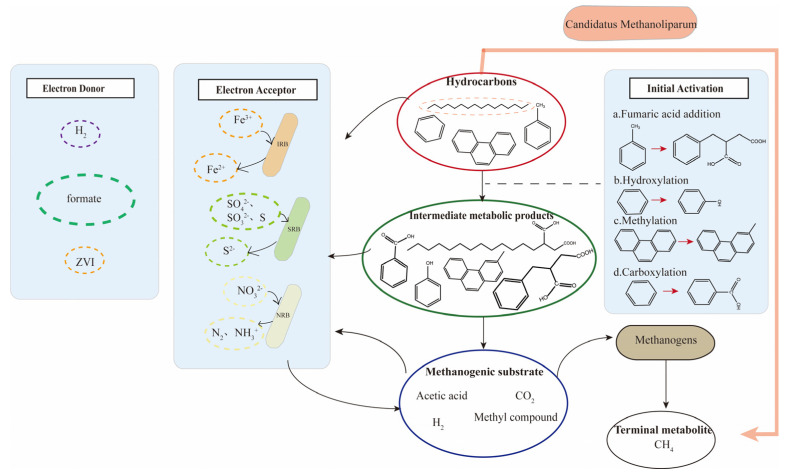
Overview of crude oil gasification metabolic pathways.

Reservoir crude oil primarily consists of saturated hydrocarbons, aromatic hydrocarbons, asphalt, and other non-hydrocarbons, with over 80% being hydrocarbons [73,74]. Microorganisms degrade the components of crude oil aerobically and anaerobically, gradually transforming them from long-chain complex compounds to small-molecule carbon-containing compounds. Aerobic degradation, involving both facultative anaerobic and aerobic microorganisms, breaks down petroleum hydrocarbons into small molecules like carbon dioxide through the action of oxygenase enzymes, utilizing molecular oxygen as the electron acceptor [75]. In contrast to aerobic degradation, anaerobic degradation is slower and continuous. Facultative aerobic bacteria and anaerobic bacteria use nitrate, sulfate, carbon dioxide, etc. as electron acceptors, ultimately converting petroleum hydrocarbons to CH_4_ [76,77]. Anaerobic degradation is the predominant process in the gasification of crude oil in reservoirs, typically encompassing multiple reaction steps, including the initiation of activation, fermentation, hydrolysis, anaerobic oxidation, and methanogenesis [78], as depicted in Figure 4. This paper specifically concentrates on the anaerobic degradation of petroleum hydrocarbons and the methanogenic pathway.

### 3.1. Mechanism of Anaerobic Degradation Activation in Reservoir Crude Oil

Biegert et al. [79], Rabus et al. [80], and Aitken et al. [35] discovered and proposed the fumaric acid-addition pathway. Kniemeyer et al. [81] clarified that the core of the process involves the addition of fumaric acid to the sub-terminal carbons of alkanes or the alkyl side chain of aromatic compounds through isotopic labeling of intermediate metabolites. Table 4 lists the fumarate addition pathways of different substrates. Mbading et al. [82], Gieg et al. [83], and others have experimentally demonstrated that alkanes can produce 1-methyl alkyl succinic acid through the addition of fumaric acid to the subterminal carbon under various conditions and then undergo carbon backbone rearrangement, isomerization, decarboxylation, and multistep β-oxidation to achieve activated degradation. Herath et al. [84], Yoshikawa et al. [85], Alegbeleye et al. [86], and others found that substituted aromatic hydrocarbons have a fumaric acid-addition pathway similar to that of alkanes. For example, in the case of m-xylene, the methyl group on the benzene ring of m-xylene undergoes an addition reaction with fumaric acid to produce phenylmethyl succinate, which is then oxidatively dehydrogenated through β-oxidation to form benzoyl coenzyme A, entering the central metabolic pathway [84]. Researchers such as Boll [87] and Abu [88] have experimentally confirmed that, despite the high-bond energies making benzene itself difficult to degrade, it can be converted into substituted aromatics under various reducing conditions and subsequently participate in the fumaric acid addition.

Besides the typical fumaric acid-addition pathway, the initial activation of crude oil components also involves hydroxylation, carboxylation, and methylation pathways. The hydroxylation pathway is relatively underreported, with its main targets being individually substituted aromatics and alkanes, such as ethylbenzene and propylbenzene. For instance, Heider et al. [95] discovered that ethylbenzene dehydrogenase and its encoding gene, *ebdABC*, are linked to the initiation of anaerobic degradation of ethylbenzene and propylbenzene. This enzyme catalyzes the hydroxylation of the alkyl side chains of ethylbenzene and propylbenzene in anaerobic environments, resulting in the production of 1-phenylethanol and 1-phenylpropanol. Aeckersberg et al. [37] identified the encoding gene, *ahy ABC*, in the strain *Desulfococcusoleovorans Hxd3*, which was isolated from oil and water samples due to its analogous function to the encoding gene *ebdABC*. Table 5 lists the hydroxylation pathways of different substrates.

Different from the fumarate-addition pathway and hydroxylation pathway, carboxylation and methylation pathways are primarily suitable for non-substituted aromatic compounds such as benzene, naphthalene, and phenanthrene, as well as a few substituted aromatic compounds like 2-methylnaphthalene and phenol. Abu [88] and Ye [104] et al. have demonstrated that under specific conditions, benzene and naphthalene can be directly carboxylated to form benzoate or naphthalene ester through the assumed carboxylase catalyst (Abc) for degradation. Additionally, Bergmann et al. [105] discovered that sulfate-reducing bacteria N47 could degrade substituted aromatic compounds like 2-methylnaphthalene and phenol via a carboxylation initiation reaction, with the gene sequence of naphthylkylase A reductase (NCR) only found in the anaerobic process of sulfate-reducing naphthalene degradation [106]. Furthermore, Safinowski [107] and Tasi [108] found that in a medium containing carbonate as an activator, methylation reactions led to the production of 2-methylnaphthalene or 2-methyl phenanthrene from naphthalene, phenanthrene, and other aromatic hydrocarbons. This discovery enriched our understanding of the initial activation pathway for aromatic hydrocarbons. Table 6 lists the carboxylation and methylation pathways of different substrates.

**Figure 5:**
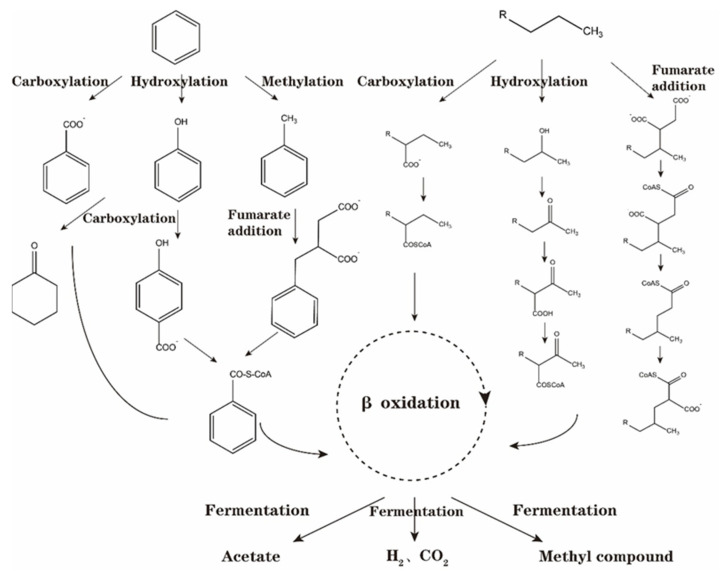
Metabolic processes of benzene and alkane under different activation pathways of anaerobic degradation initiation.

In conclusion, the initial activation metabolism of crude oil hydrocarbon components under anaerobic conditions primarily involves fumaric acid addition, hydroxylation, carboxylation, and methylation. Subsequently, the initial activation product undergoes a series of changes, such as carbon rearrangement and decarboxylation, transforming into alkyl succinic acid of fatty acids and aromatic hydrocarbons and further enters the central metabolic pathway through β oxidation to metabolize acetic acid, methyl compounds, hydrogen, and carbon dioxide. Using benzene as an example, it undergoes anaerobic activation and degradation to enter the central metabolic process of benzoyl-coA, producing acetic acid, methyl compounds, hydrogen, and carbon dioxide, as illustrated in Figure 5.

### 3.2. Mechanism of Methanogenesis

**Figure 6:**
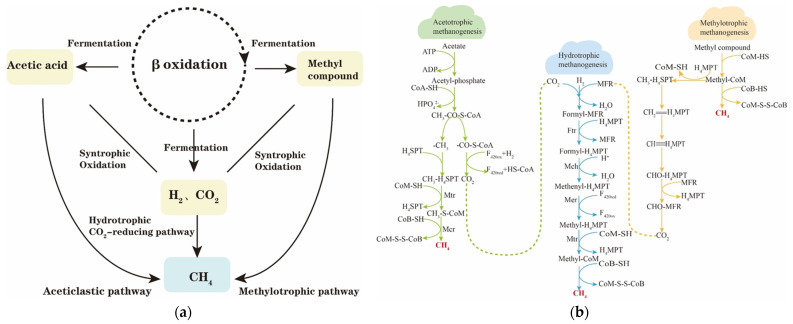
(**a**) Terminal metabolism process of crude oil gasification; (**b**) overview of the main methanogenic metabolic pathways.

Petroleum hydrocarbons undergo a series of anaerobic degradation reactions, gradually transforming into volatile fatty acids, soluble degradants, acetic acid, carbon dioxide, and hydrogen. Ultimately, under methanogenic conditions, the anaerobic degradation metabolites, including acetic acid, methyl compounds, hydrogen, and carbon dioxide, are converted into methane by methanogenic archaea, as shown in Figure 6. The methanogenic metabolic pathways can be broadly categorized into two groups: the reduction of carbon dioxide to produce methane, and the decomposition of small molecules such as acetic acid and methyl compounds into methane. These pathways are further subdivided into three core methanogenic pathways: the hydrogen–trophic pathway, the acetotrophic pathway, and the methyl–trophic pathway. Table 7 illustrates the major methanogenic metabolic pathways and the corresponding Gibbs energy of the reaction. Additionally, other metabolic pathways may be activated under specific conditions. For instance, in environments with high temperatures and low pH, an inter-interacting acetic acid oxidation pathway is stimulated, wherein acetic acid is oxidized by inter-interacting acetic acid oxidizing bacteria to produce hydrogen and carbon dioxide, ultimately leading to methane production by hydrotrophic methanogens. The direct acetic acid oxidation pathway is activated at high concentrations of CO_2_.

**Figure 7:**
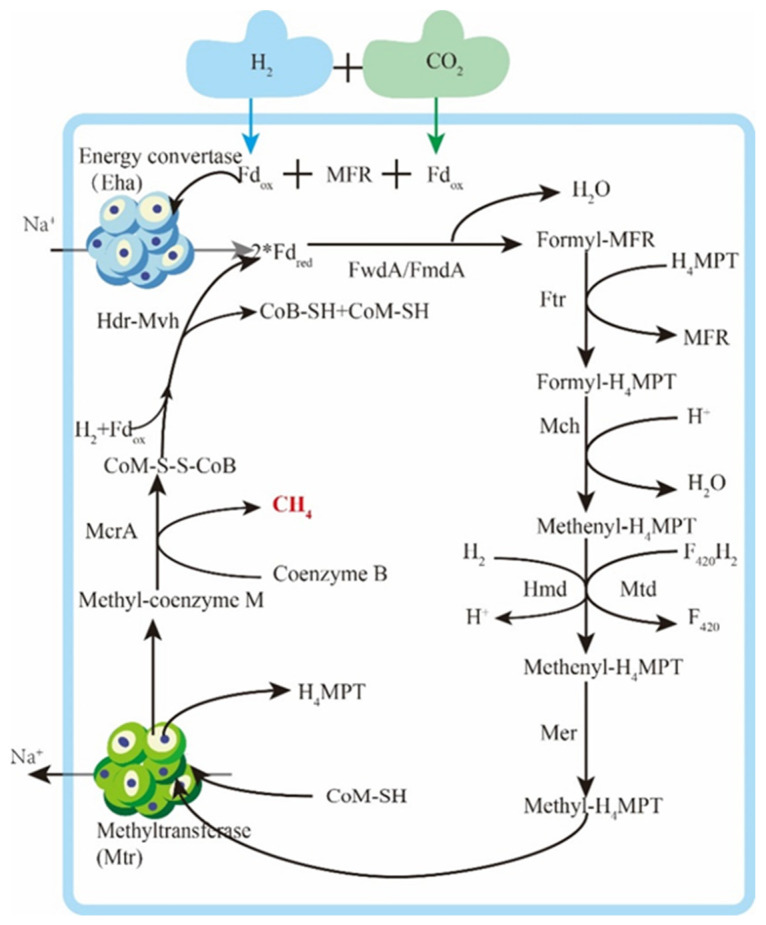
Hydrotrophication methanogenic metabolic pathway.

In the hydrogenotrophic methanogenesis reaction, H_2_ reduces CO_2_ to methane. This process involves the transfer of carbon units between coenzymes and dehydration condensation reactions, as depicted in Figure 7. Electron transfer is mediated by the deazaflavin coenzyme F_420_ or ferredoxin (Fd), while various hydrogenases and/or dehydrogenases conduct the reduction of these electron carriers. The realization of the hydrotropic pathway heavily relies on electron transfer, and the current widely recognized electron bifurcation theory based on flavin and the Eha hypothesis of energy conversion hydrogenase play crucial roles. Herrmann et al. [116] proposed the electron bifurcation theory based on flavin, in which flavin protein splits electron pairs into a low-potential electron and a high-potential electron. This process achieves low-potential electron-reducing Fd under the mediation of ferredoxin (Fd) and simultaneously releases energy through the high-potential electron reduction of NADH. Lie et al. [117] verified the electron bifurcation hypothesis based on flavin through experiments and found that the energy-converting hydrogenase Eha, as a means to provide electrons to formylmethanofuran dehydrogenase, can supplement the electron bifurcation in a non-hybrid manner.

**Figure 8:**
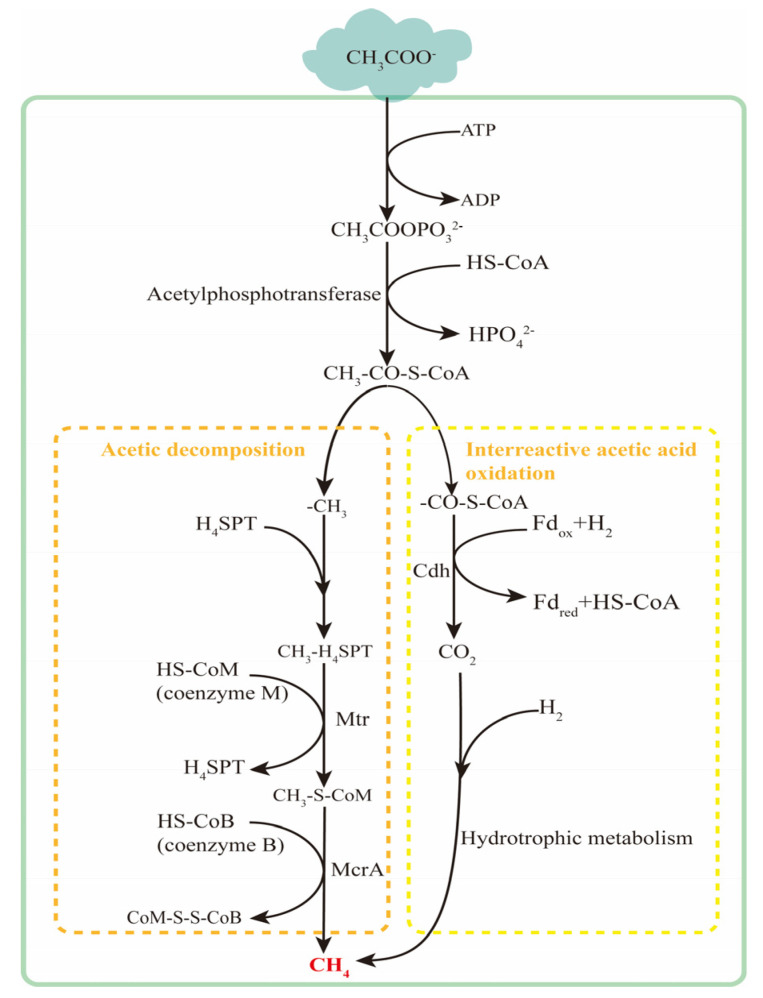
Acetotrophic methanogenic metabolic pathway.

There are two metabolic pathways of methanogenesis using acetic acid as a substrate. One is the direct reduction pathway of acetic acid, involving the direct reduction of methyl in acetic acid to methane, as shown in Figure 8. The second is the interacting acetic acid-oxidation pathway, where acetic acid is oxidized by inter-interacting acetic acid-oxidizing bacteria to produce hydrogen and carbon dioxide, ultimately leading to methane production by hydrogenotrophic methanogens. Both pathways compete for acetic acid as the substrate. Dolfing et al. [118], based on physicochemical theory, analyzed that under high-temperature and low-pH conditions, the reaction of acetic acid mutual oxidation, combined with the h hydrotrophic methanogenesis reaction, is more thermodynamically favorable. High concentrations of carbon dioxide may stimulate the acetic acid-reduction pathway. Studies involving animals or humans, and other research requiring ethical approval, must specify the approving authority and the corresponding ethical-approval code. The two-step reaction involved in the reciprocal oxidation of acetic acid is shown in Equations (1) and (2). The overall reaction for the production of methane from acetic acid is shown in Equation (3).

(1) $$CH_{3}COOH+2H_{2}O\to 2{CO}_{2}+H_{2},$$

(2) $$4H_{2}+{CO}_{2}\to {CH}_{4}+2H_{2}O,$$

(3) $$CH_{3}COOH\to {CH}_{4}+{CO}_{2}$$

**Figure 9:**
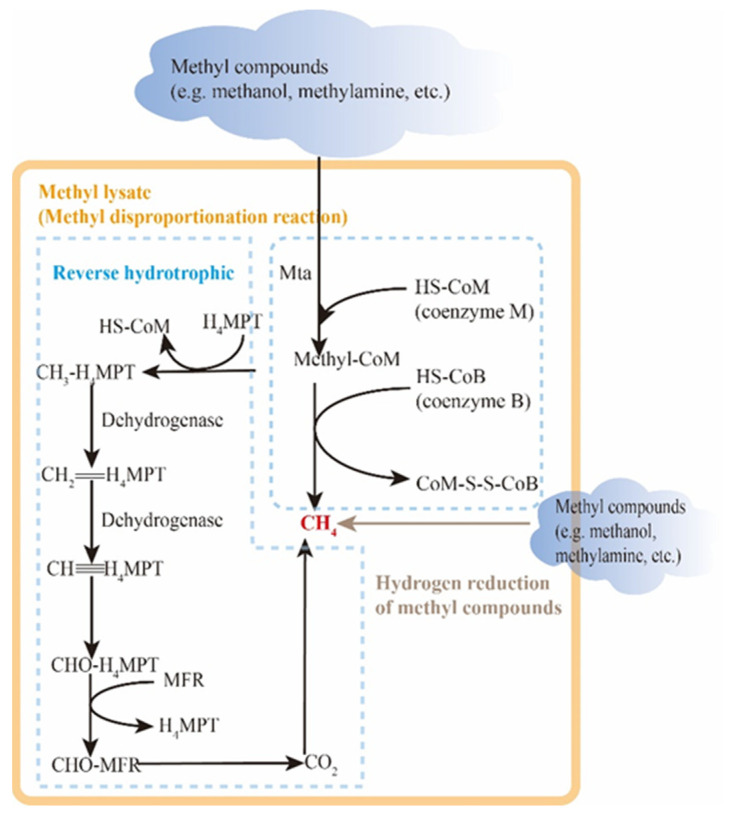
Methylotrophic methanogenesis pathway.

The methylotrophic pathway mainly utilizes methyl compounds such as methanol and trimethylamine as substrates, as depicted in Figure 9. Since methyl species are not as widely distributed in nature as acetic acid and carbon dioxide, the methylotrophic pathway is primarily found in highly mineralized environments such as salt lakes. The methylotrophic pathway can be further categorized into the methyl lyase type and the H_2_-reduced methyl compound type. The term “methyl cleavage type” refers to the production of methane through the self-disproportionation reaction of methyl compounds. Duszenko et al. [119] discovered that extreme halophilic methanogens, such as methananotronarchaea, utilize C1 methyl compounds as electron acceptors and formate or hydrogen as electron donors. Through the action of methyltransferase, one out of every four methyl compounds is oxidized to CO_2_ via the reverse hydrotrophic pathway, while the remaining three are reduced to methane, with the loss of electrons being internally resolved. The distinction between H_2_ reduction of methyl compounds lies in the fact that, during H_2_ reduction of methyl compounds, methyl compounds solely function as electron acceptors and are directly reduced to methane, making the process more efficient and straightforward. Yang et al. [120] demonstrated that in the anaerobic culture system of carbon dioxide and formic acid in reservoir production fluid, both the symbiotic oxidation of formic acid linked to hydrotrophic methanogenesis and the direct cracking of formic acid methanogenesis occur.

In addition to the aforementioned approaches, Zhou et al. [121] comprehensively utilized carbon isotope labeling, metagenomic (transcriptomic) analysis, and other technologies to investigate the novel methanogenic archaea *Candidatus Methanoliparum*. Their research confirmed that this archaeon has the capability to autonomously degrade long-chain alkanes and produce methane, challenging the conventional understanding of methanogenic metabolism within bacterial groups. Furthermore, they identified a new methane-producing pathway, the long-chain alkane-degradation pathway.

## 4. Assessment of the Significance of Various Factors Influencing the Efficiency of the Reservoir Crude Oil Gasification Process

Currently, research on crude oil gasification is predominantly focused on the laboratory stage, and the limited gasification efficiency hinders its widespread application in engineering practice. Therefore, there is a need for a comprehensive understanding of the key factors influencing the efficiency of crude oil gasification. Building upon prior research, this paper provides a summary and analysis of the factors affecting crude oil gasification efficiency and proposes potential methods for evaluating their significance by integrating statistical analysis and machine-learning algorithms.

### 4.1. Factors Affecting the Efficiency of Crude Oil Gasification

The microbial oil gasification process primarily occurs at the oil–water interface of formations or in the micro-water environment of water-in-oil emulsion droplets. The findings indicate that the physical properties of the two phases involved at the oil–water interface and the contact area of the two phases somewhat influence gasification efficiency. The physical properties of the water phase, including the pH value, salinity, trace-element content, ammonia content, total nitrogen content, ammonia-to-alkalinity ratio, and concentration of inhibitors that predominantly impact the gasification process through microbial activity and metabolic pathways. Under normal conditions, the optimal pH for in-situ methanogenic bacteria in oil reservoirs is approximately 7. Chen et al. [122] observed that the appropriate initial pH value can enhance microbial activity and reduce the residence period, thereby increasing methane production. Salinity, as a significant measure of the salt content of formation water, to some extent affects the selection of methane-producing pathways. Waldron et al. [123] found that low salinity mainly favors hydrogen and acetic acid nutrient types, while high salinity favors methyl nutrient types. Additionally, trace elements such as Fe, Co, and Ni are closely associated with the enzymatic reaction rate of key enzymes in the gasification process, indirectly affecting the gasification rate. The physical properties of oil reservoirs, including porosity, permeability, saturation, capillary force, and wettability, also influence the growth and metabolism of microorganisms, thereby affecting crude oil gasification. In general, higher rock porosity provides more space for microbial growth, facilitating aggregation and growth. Furthermore, the greater the hydrophilicity of the rock surface, the stronger the adsorption of nutrients, including organic acids, which promote microbial metabolism and indirectly impact gasification. Additionally, the contact area of the oil–water phase directly affects the uptake and utilization efficiency of oil hydrocarbon components and the formation of water nutrients by bacteria.

The efficiency of crude oil gasification heavily relies on the substrate supply and microbial utilization rate. The primary substrates for crude oil gasification encompass the carbon source, electron acceptor, and electron donor. Some of the carbon originates from the hydrocarbon components inside the reservoirs, while the rest comes from external sources such as carbon dioxide or bicarbonate. The reservoir’s hydrocarbon components include short-chain hydrocarbons, long-chain normal alkanes, long-chain isomeric alkanes, aromatic hydrocarbons, cycloalkanes, and asphaltenes, among others. According to Table 8, the methane production efficiency of different hydrocarbon mechanisms indicates that the gasification efficiency of alkanes is markedly greater than that of asphaltenes. Furthermore, Mayumi and others observed that the injection of carbon dioxide as an exogenous carbon source accelerated the methane-production time [114]. Approximately 50% of the methane generated by gasification in the presence of hexadecane came from exogenous bicarbonate, leading to a rise in methane yield from 78.36 μmol to 147 μmol, and the methane-generation rate rose from 0.10 μmol/d to 0.16 μmol/d [124], which promoted methane formation to a certain extent.

Additionally, the gasification of crude oil involves a variety of electron acceptors, including Mn^4+^, NO_3_^−^, Fe^3+^, SO_4_^2−^, and CO_2_, in response to the decrease in REDOX potential. The participation of electron acceptors aids in consuming the organic acids produced in the degradation process of crude oil and maintaining a dynamic equilibrium between the organic acids produced by the degradation of crude oil components and those broken down through subsequent oxidation. Simultaneously, due to the competition for substrates among sulfate-reducing bacteria, nitrate-reducing bacteria, and methane-producing bacteria, excessive NO_3_^−^ and SO_4_^2−^ directly inhibit the formation of methane. Therefore, maintaining an appropriate electron acceptor ratio is crucial for the continuation of the initial degradation and methanogenesis process, as well as for the impact of methanogenesis. In comparison to abundant electron acceptors, the types of electron donors in oil reservoirs are scarce, with hydrogen and formate being the main contributors to the process of crude oil gasification. The environmental concentration of hydrogen is crucial as the direct electron donor for methane production, influencing the proportion of various pathways in the methane production process. Transcriptomic [129,130] and proteomic [131,132] studies have shown that the methanogenic pathway is heavily influenced by H_2_. Dolfing et al. [118] found that the partial pressure of H_2_, in most oil reservoirs, is typically maintained at a low level (<10^−2^ atm) or is sometimes undetectable, so it is necessary to introduce or stimulate H_2_-producing bacteria when constructing the crude oil degradation methanogenic bacteria in simulated in situ gasification experiments. Additionally, there are limited studies on alternative electron donors. For instance, ZVI (zero-valent iron) can be utilized as an alternative electron donor. Ma et al. [128] used a ZVI-modified substrate as an alternative electron donor. Scanning Electron Microscopy–Energy-Dispersive X-ray Spectroscopy (SEM-EDS) and hydrogen mass balance results proved that ZVI was feasible, and the rate of reduction was improved. An adequate amount of electron acceptors and donors is crucial to achieve efficient electron transport of microorganisms, ensuring the continuous and stable process of initial degradation and methanogenesis.

Besides the environmental and substrate factors mentioned above, the oil gasification process encompasses a variety of enzymes, which determine the reaction rate and direction to some extent. The primary activation process of crude oil anaerobic degradation primarily involves fumarate addition and exhibits specific enzymes and coding genes that catalyze different substrates. The most common ones are alkyl succinate synthetase and methyl succinate synthetase, which catalyze alkanes. Alkyl succinate synthetase is mainly used to catalyze the degradation of long-chain normal alkanes, while the methyl–alkyl succinate synthetase is primarily used for anaerobic degradation of normal alkanes in the C6-to-C8 range. The associated genes for their α-subunits are *ass ABC* [133] and *masD* [134], respectively. Meanwhile, with the development of whole-gene technology, Zhu et al. [135] hypothesized that the coding genes *pfl D* and *pfl C* also have functions similar to *ass ABC*. Additionally, phenyl methyl succinic synthetase is responsible for catalyzing the degradation of aromatic substituents such as toluene, xylene, and ethylbenzene, whose encoding gene is *bssABC*, while the anaerobic degradation of naphthalene and methylnaphthalene of PAHs depends on naphthalene methyl succinic synthetase, whose encoding gene is nms [106].

Methyl-coenzyme M is involved in the final step of methanogenesis, catalyzing the reaction of Coenzyme B (CoB-SH) to produce the final product CH_4_. Grabarse and colleagues compared the crystal structure and gene sequence of the methyl-coenzyme M reductase protein of different methanogenic organisms and found that they were closely related [136]. Meanwhile, through an analysis of the crystal structure of the protein, it was found that they contained two forms, MCR-Ⅰ and MCR-Ⅱ. MCR-Ⅰ is encoded by *mcrBDCGA*, while the proteins in Methanococcales and Methanobacteriales are different from MCR-Ⅰ and encoded by *mrtBDGA* [137]. To further clarify the relationship between coding genes and methane-production efficiency, scientists found that the copy number of the mcrA gene in the hydrogen–trophic pathway was significantly positively correlated with the methane-production efficiency through genetic library and quantitative PCR amplification, while the correlation between the transcription number of *mcrA* and the activity of specific methanogenic bacteria was not obvious [138].

In addition to methyl-coenzyme M, there are also key enzymes and coding gene clusters in the three major methanogenic core pathways. A variety of hydrogenases and dehydrogenases are involved in the electron gain and loss of carbon dioxide reduction through the hydrogenotrophic pathway, including energy-converting hydrogenase (Ech), F420-reducing hydrogenase (Frh), tungsten-containing methanofuran dehydrogenase (Fwd), and molybdenum-containing methanofuran dehydrogenase (Fmd). Among them, the structure of molybdenum formylmethanofuran dehydrogenase (Fmd) and its related gene cluster *FmdABC* are very similar to *FwdABCD* in function and coding sequence [139]. The central enzyme of the acetoclastic pathway is carbon monoxide dehydrogenase, which, by structural analysis, is found to exist in the form of a dimer composed of L, M, and S triacyl groups, and its core metabolic gene is *CdhABGDE*. A comparison of the abundance of all key genes shows that the *cdhD* gene has a low abundance and exists in all acetogenic methanogenic processes. In the methylotrophic pathway, coenzyme M methyltransferase (Mtr) plays a key role that is responsible for transferring methyl from methanol and methylamine to coenzyme M to generate methyl-Coenzyme M (CH_3_-S-CoM). Sequencing found that its main component, subunit 8, was encoded by *MtrECDBAFGH* [140]. Additionally, Hedderich et al. [141] and Zhou et al. [33] found that *mta*, *mtb*, *mts*, *mtq*, *mtt, mtm*, and other coding genes exist in the genome of methanogenic archaea. In short, with the development of cryo-electron microscopy and metagenomic technology, researchers have gained insights into the functional genes of key enzymes based on the analysis of the structural characteristics of key enzyme proteins, providing new ideas for discovering new metabolic pathways in the future and improving methane production from oil reservoirs through protein-transformation engineering.

### 4.2. Multivariate Importance Evaluation Methods for Enhancing Oil Reservoir Gasification Efficiency

**Figure 10:**
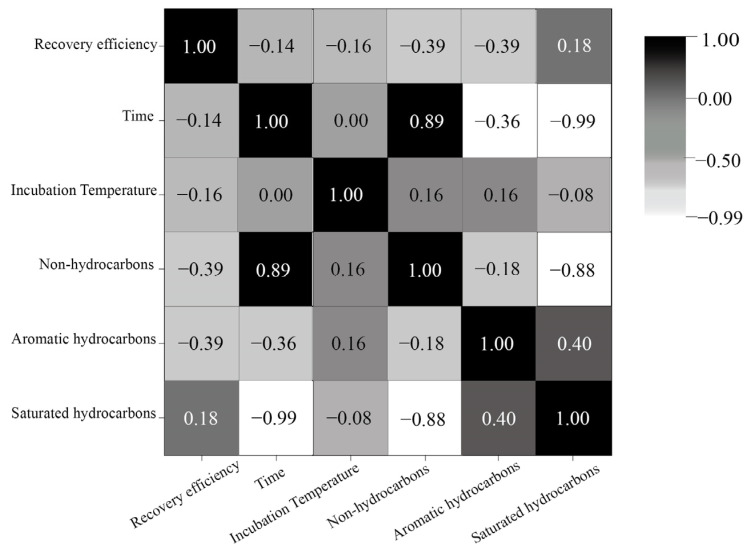
Spearman correlation analysis heat map of different crude oil components at different temperatures and times.

Oil reservoir gasification efficiency is influenced by various factors, including environmental, substrate, and biological variables, as shown in Table 9. A precise evaluation of the relative significance of each factor aids researchers in identifying key regulatory targets, streamlining experimental design, and enhancing time efficiency. To quantitatively assess the relative importance of these factors, Darlingto [142], Johnson [143], and their colleagues introduced evaluation metrics such as the correlation coefficient, partial correlation coefficient, standardized regression coefficient, covariance, dominance analysis, and relative weight. Currently, simple correlation analyses such as the Pearson [144], Spearman [145], and Kendall correlation analyses [146], as well as complex nonlinear analysis methods, including multiple correspondence analysis, classified nonlinear principal component analysis, nonlinear typical analysis, and gray correlation analysis [147], are predominantly utilized for experimental results with relatively small sample sizes. These methodologies have found application in engineering-problem domains, such as the prediction of initial productivity in oil wells. Song Xuanyi and colleagues employed Pearson correlation analysis to construct a correlation matrix for 10 characteristic parameters, including porosity and permeability and subsequently assessed the correlation of each parameter. Their findings revealed a strong linear relationship between porosity and permeability, as well as a correlation between oil-layer thickness, perforation-section thickness, and fracturing light. This suggests the potential for simplified grouping of multiple variables [148]. In this study, utilizing experimental data from Cheng et al. [4] regarding the degradation of various crude oil components at different temperatures and durations, a correlation analysis heat map was generated based on the Spearman correlation coefficient, as depicted in Figure 10. The figure illustrates that saturated hydrocarbons and non-hydrocarbon substances exhibit a significantly higher time dependency compared to aromatic hydrocarbons. Furthermore, a notable correlation between saturated hydrocarbons and non-hydrocarbon substances suggests that saturated hydrocarbons play a pivotal role in enhancing crude oil degradation efficiency.

**Figure 11:**
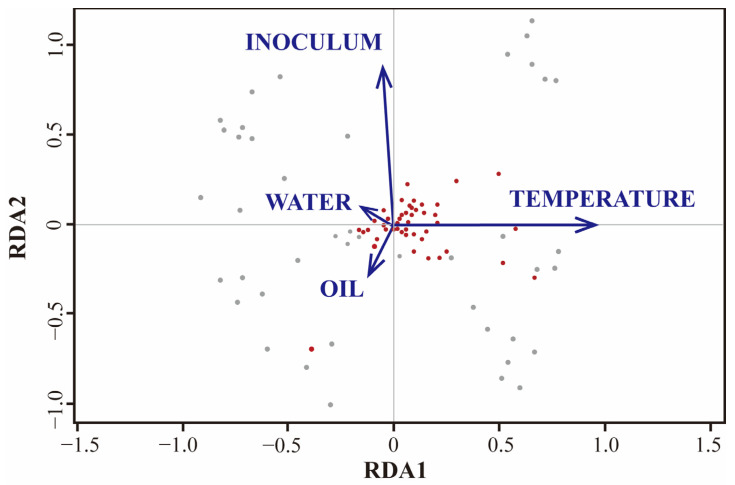
Two-line plot of microbial community redundancy analysis (RDA) under oil, temperature, nutrients, and initial community constraints [149]. The red and gray dots represent the two treatments.

Moreover, the gasification process of crude oil relies on the involvement of a microbial community and associated biological enzymes. In their study, Liu et al. [149] analyzed the microbial community using non-metric multidimensional scaling (NMDS) and subsequently subjected it to similarity analysis (ANOSIM). Their findings revealed that various factors, particularly temperature and the initial community, were the primary driving forces influencing the microbial community, as depicted in Figure 11. Notably, oils and nutrients were found to play a secondary role. However, this analysis solely pertains to the structure of the microbial community. To accurately evaluate the variables of oil gasification, it becomes imperative to consider the enzymes and multiple functional genes involved in the intricate nature of biological structures and genetic polymorphism results in a comprehensive crude oil gasification dataset comprising hundreds or thousands of feature parameters. Depending solely on traditional feature correlation-evaluation methods for analysis becomes time-consuming and laborious. Faced with extensive datasets, machine-learning algorithm modeling demonstrates a superior performance compared to traditional correlation analysis methods. Common machine-learning algorithms encompass linear regression, logistic regression, linear discriminant analysis, naive Bayes, KNN, artificial neural networks, and random forest, among others, as listed in Table 10. Selecting the suitable algorithm based on the dataset’s characteristics and the intended implementation conditions is crucial. Table 5 provides a summary of common machine-learning algorithms, along with their respective advantages and disadvantages. By randomly selecting different variables, assigning random values to each predictor, and calculating the error rate, one can select a feature variable and introduce random noise to the sample’s feature variable. This enables the comparison of the variance change predicted by the model after the value is randomly replaced [150]. The proportion of the model’s variance increase serves as a criterion for assessing the importance of variables, revealing the relationship between each variable and the oil gasification efficiency in the reservoir. A higher error in the overall model after replacement indicates a stronger importance of the predictor variable. Kacalla and colleagues constructed a database comprising 32 variables from 93 reservoirs on the Norwegian continental shelf. They employed the random forest algorithm to develop three prediction models and investigated the impact of various input variables on recovery rate and production. Their analysis revealed that when predicting the oil storage rate, the most crucial variables were associated with the size of the oilfield, including cumulative oil production, number of wells, oil production (OIP), and rock volume [151]. In a separate study, Yu et al. [152] utilized the random forest (RF) machine-learning algorithm to explore the factors influencing the high and stable production of a single well within a specific well group in the Junggar Basin. Their findings indicated that reservoir quality and oil saturation are the primary controlling factors, establishing the fundamental basis for achieving high and stable oil production [153]. These studies demonstrate the significant advantages of the random forest model in evaluating the importance of multiple variables in large datasets. Consequently, its application in assessing the multivariate importance of the crude oil gasification process is anticipated to enable the precise regulation of the gasification process.

## 5. Opportunities and Challenges of Microbial Assisted Crude Oil Gasification in Practical Applications

The utilization of microbial assistance in the gasification of crude oil presents both opportunities and challenges in practical applications. This pioneering approach involves microbial assistance to enhance the gasification process of refractory crude oil in reservoirs, thereby enhancing the utilization of petroleum resources and promoting the sustainable development of the petroleum industry. In addition, under either anaerobic or aerobic conditions microorganisms reveal their large potential in biodegradation of contaminants [148], especially crude oil-contaminated wastewater [156], crude oil-polluted soil [157], sewage sludge, composition [158], etc.

The successful implementation of the technology encounters numerous challenges. To address the practical requirements of engineering applications, the technical feasibility of microbial-assisted crude oil gasification under reservoir geothermal conditions must be prioritized. This necessitates researchers to comprehend the distinctions between simulated laboratory operating conditions and the genuine engineering temperature and pressure conditions. Additionally, to fulfill the demands of large-scale production, continuous testing and optimization of the reaction are essential to enhance the efficiency of crude oil gasification and achieve cost-effective process control. Therefore, while microbial-assisted crude oil gasification is expected to drive sustainable development in the oil industry, these complexities also need to be carefully considered to effectively integrate it into practical situations.

Laboratory simulation experiments have validated the gasification of crude oil by microorganisms. Thiagarajan et al. have further corroborated the microbial-assisted crude oil gasification process, yielding methane in deepwater oil reservoirs in the Gulf of Mexico [151]. This confirmation underscores the technical viability of microbial-assisted crude oil gasification initiatives. Currently, most projects in this realm remain at the laboratory stage, constrained to some extent by the intricate reservoir conditions. Bridging the gap between real geological conditions (<200 °C) and laboratory settings (30–110 °C) is paramount for industrial implementation [153]. While existing experiments predominantly employ single-factor variable methods, focusing on simplified temperature, pressure, and crude oil compositions, the actual reservoir conditions exhibit significant temperature and pressure variations due to depth, coupled with more complex crude oil compositions. These factors influence the anaerobic degradation rate of crude oil and impede the reliable extrapolation of experimental outcomes to practical engineering applications. Due to the complexity of practical applications, the multi-variable coupling relationship should be fully considered before engineering practice, and computational fluid mechanics and quantum chemistry calculation methods [153] should be combined to simulate the microbial assisted crude oil gasification process in real reservoirs.

Currently, the primary challenge in crude oil gasification is the low efficiency of the process. To meet the demands of large-scale production, the continual enhancement of expertise in microbial-assisted metabolic for crude oil gasification is essential. This involves seeking more efficient and direct metabolic pathways, optimizing the microbial flora combination to maximize the synergistic effect of crude oil-degrading bacteria. Furthermore, it is crucial to pinpoint the key factors influencing gasification efficiency and enhance it by introducing exogenous nutrients and alternative electron donors.

Additionally, engineering practices face a range of risks, including environmental hazards and quality and safety risks linked to diverse integrated technologies. The primary focus of crude oil gasification lies in abandoned well groups within depleted reservoirs. Setting up a strong oil and gas field infrastructure along with a thorough risk-assessment system lays the groundwork for repurposing abandoned reservoirs as natural gas-storage sites [159]. Furthermore, refurbishing old wells using methods like gravel packing can notably improve well-safety performance and reduce the risk of leaks.

## 6. Conclusions and Prospect

In light of the “2030” carbon peak and “2060” carbon neutrality commitment, carbon capture, utilization, and storage (CCUS) technology holds significant potential for application and development [160]. The China Carbon Dioxide Capture, Utilization, and Storage (CCUS) Annual Report 2021 underscores that CCUS technology stands as the sole option for decarbonizing fossil energy. The biodegradation of crude oil components by in situ microorganisms can effectively enhance resource utilization and simultaneously convert exogenous carbon dioxide into methane, which can be utilized as natural gas or stored as a strategic resource, holding substantial implications for economic development and carbon emission reduction.

This study provides a comprehensive overview of the primary metabolic pathways and microbial communities involved in oil reservoir gasification, along with an exploration of the potential biological prospects of oil gasification in diverse environments. Numerous environmental factors, including the temperature, contact area, substrate, nutrients, and electron acceptor, directly impact the efficiency of crude oil gasification. However, among these factors, the assessment of importance indicates that the proportion of crude oil components and the contact area between microorganisms and petroleum hydrocarbons are more manageable. Thus, under suitable conditions, implementing substrate regulation and developing new biocompatible surfactants to enhance the contact surface between petroleum hydrocarbons and microorganisms may serve as a potential approach to effectively improve the efficiency of crude oil gasification. Further research is essential to understand the steps and limitations of the speed of crude oil gasification in the future, and the utilization of big data and the random forest model to assess the importance of multiple variables and introduce evaluation indicators for quantitatively measuring the relative significance of various factors in the gasification process. Unraveling the mechanisms behind these correlations may pave the way for addressing the challenge of crude oil gasification efficiency and realizing the large-scale application of crude oil gasification facilitated by microorganisms in the future.

In addition, 16S rRNA and metagenomic technologies were utilized to investigate the microbial community involved in crude oil gasification. It was discovered that addressing the challenge of crude oil gasification efficiency may necessitate the cumulative synergistic symbiosis among different types of hydrocarbon-degrading bacteria, including fermentation bacteria, sulfate-reducing bacteria, and methanogens. Consequently, the development of appropriate microbial consortia can enhance the utilization rate of recalcitrant crude oil components and facilitate the timely and efficient conversion of intermediate metabolites into end products. In the future, there is a need to expand and enrich the repertoire of functional bacterial resources through high-throughput screening methods, uncover untapped reservoirs of petroleum hydrocarbon-degrading bacteria, and further refine the strategy for constructing artificial microbial communities.

Based on this foundation, potential undiscovered pathways for the degradation of crude oil components by unknown bacterial populations may exist, warranting the urgent advancement and enhancement of microbial-isolation technologies. This involves integrating meta-transcriptomic, metagenomic technologies, and low-throughput biomarker monitoring to analyze and identify potentially more efficient pathways for crude oil metabolism.

## Figures and Tables

**Table 1:** Bacterial flora for crude oil degradation in oil reservoirs.

Metabolic Process	Classes of Bacteria	Representative Strain	Refs.
Hydrocarbon degradation	Fermenting bacteria	*Thermococcus*, *Thermoanueroubacter*, *Haloanaerobium*	[20,21,22]
Hydrocarbon degradation	Hydrocarbon oxidizing bacteria	*Pseudomonas*, *Bacillus*, *Acinetobacter*	[23,24,25]
Hydrocarbon degradation	Nitrate-reducing bacteria	*Deferribacteres, Firmicutes*, β-*proteobacteria*	[26,27]
Hydrocarbon degradation	Sulfate-reducing bacteria	*Firmicutes*, δ-*proteobacteria*, *Thermodesulfbacterium*	[28,29,30]
Hydrocarbon degradation	Iron-reducing bacteria	*Deferribacteres*, *Firmicutes Metallireducens,*	[31,32]
Methanogenic	Methanogens	*Methanococcales*, *Methanomicrobiales*, *Methanobacteriales*	[33,34,35]

**Table 2:** Bacteria degraded by crude oil in different reservoirs.

Origin of the Inoculum	Substrate, Temperature/°C	Bacterial Flora	Refs.
Shengli oil field, PR China	C_15_–C_20_ alkanes, 55	*Firmicutes*, *Thermodesulfobiaceae*, *Thermotogaceae*, Nitrospiraceae, Dictyoglomaceae, Archaeoglobales	[42,43,44]
Dagang oil field, PR China	Oil, 30	*Pseudomonas*, *Smithella*, *Syntrophorhabdus*, *Desulfobulbus*, *Methanosaeta*, *Thermoplasma*	[45,46,47]
Medicine Hat, Canada	Oil, 33	*Smithella*, *Pseudomonas*, *Methanosaeta*, *Methanoculleus*, *Methanobacterium*	[48,49,50]
Mildred Lake Settling Basin, Canada	C_14_–C_18_ alkanes, 20	*Syntrophus*, *Desulfuromonas*, *Desulfobacter*, *Methanosaeta*, *Methanoculleus*	[51,52,53]

**Table 3:** Methanogenic archaea and their metabolic substrates.

Orders	Representatives	Degradation Substrate	Refs.
Methanobacteriales	*Methanobacterium* *Methanobrevibacter* *Methanothermobacter* *Methanothermus*	Hydrogen, carbon dioxide, formates, methanol	[56,57]
Methanococcales	*Methanococcus* *Methanothermococcus* *Methanocaldococcus*	Hydrogen, carbon dioxide, formates	[58,59]
Methanomicrobiales	*Methanomicrobium* *Methanoculleus* *Methanogenium* *Methanocalculus* *Methanospirillum* *Methanofollis* *Methanoplanus*	Hydrogen, carbon dioxide, 2-propanol, 2-butanol, acetate, 2-butanone	[60,61]
Methanosarcinales	*Methanosarcina* *Methanococcoides* *Methanohalobium* *Methanohalopholus* *Methanolobus* *Metahanosaeta*	Hydrogen, carbon dioxide, formates, acetate, methylamine	[62]
Methanopyrales	*Methanopyrus*	Hydrogen, carbon dioxide	[62]
Methanocellales	*Methanocellapaludicola* *Methanocellaarvoryzae* *Methanocellaconradii*	Hydrogen, carbon dioxide, formates	[63]
Methanomassiliicccales	*Methanomassiliicoccus* *luminyensis*	Hydrogen, methylamines, methanol	[64,65]

**Table 4:** Fumaric acid addition pathways of different substrates.

Substrate	Conditions	Process	Refs.
Alkanes	Sulfate reduction Nitrate reduction Methanogenesis	Fumarate addition→alkylsuccinate	[83,89]
Cycloalkanes	Sulfate reduction	Fumarate addition	[90]
Toluene	Sulfate reduction Nitrate reduction Iron reduction Methanogenesis	Fumarate addition→benzylsuccinate→benzoyl-CoA	[91]
Ethylbenzene	Sulfate reduction Nitrate reduction Iron reduction	Fumarate addition→benzylsuccinate→benzoyl-CoA	[85]
m-Xylene	Sulfate reduction	Fumarate addition→3-methyl benzylsuccinate→m-toluic acid	[92]
o-Xylene	Sulfate reduction	Fumarate addition→2-methyl benzylsuccinate→o-toluic acid	[85]
p-Xylene	Sulfate reduction	Fumarate addition→4-methyl benzylsuccinate→p-toluic acid	[93]
2-Methylnaphthalene	Sulfate reduction Iron reduction	Fumarate addition→2-Naphthylmethyl succinate→2-naphthyl-CoA	[94]

**Table 5:** Hydroxylation pathways of different substrates.

Substrate	Conditions	Process	Refs.
Alkanes	Sulfate reduction Nitrate reduction	Hydroxylation	[37,87,96,97]
Benzene	Methanogenesis	Hydroxylation to phenol	[98]
Ethylbenzene	Nitrate reduction	Hydroxylation→(s)-1-phenyl ethanol	[99]
Phenol	Sulfate reduction Nitrate reduction Iron reduction	Hydroxylation→catechol	[100]
Methylated phenols	Sulfate reduction	Hydroxylation→catechols	[100]
Naphthalene	Methanogenesis	Hydroxylation and ring cleavage	[101]
Phenanthrene	Sulfate reduction Nitrate reduction	Hydroxylation→2′-Hydroxypropiophenone→Phthalic acid	[102]
Phenanthrene	Sulfate reduction Nitrate reduction	Hydroxylation→4-Hydroxycinnamate→p-cresol→phenol	[102]
Benzo(a)pyrene	Nitrate reduction	Hydroxylation→4,5-dihydro benzo(a)pyrene→chrysene	[103]

**Table 6:** Carboxylation and methylation pathways of different substrates.

Substrate	Conditions	Process	Refs.
Benzene	Sulfate reduction Nitrate reduction Methanogenesis	Carboxylation→benzoate	[91]
Phenol	Sulfate reduction Nitrate reduction Iron reduction	Carboxylation →4-hydroxybenzoate	[109]
Benzoate	Sulfate reduction Nitrate reduction Iron reduction Methanogenesis	Carboxylation→benzoyl-CoA	[110]
2-Methylnaphthalene	Methanogenesis	Carboxylation→2-naphthoic acid	[104]
Biphenyl	Sulfate reduction	Carboxylation→biphenyl-4-carboxylic acid	[111]
Phenanthrene	Sulfate reduction	Carboxylation→phenanthrene-2-carboxylic acid	[112]
Naphthalene	Sulfate reduction Iron reduction	Methylation→2-methylnaphthalene	[113]
Phenanthrene	Sulfate reduction	Methylation→4-methylphenol→phenol	[108]

**Table 7:** Overview of different methane production pathways.

Condition	Methanogenesis Reaction	ΔG^θ^(25 °C)/kJ mol^−1^CH_4_	Equation	Refs.
Substrate: Hydrogen, formic acid Environment: Low, medium, and high temperature; pH 6.0~9.0 Bacteria: The vast majority of methanogens	Hydrotropic CO_2_-reducing pathway	−130.8	$4H_{2}+{{HCO}_{3}}^{-}+H^{+}\to {CH}_{4}+3H_{2}O$	[114]
Substrate: Aceticlastic Environment: Low temperature; pH 6.0~8.5 Bacteria: *Methanosarcina*, *Methanosaeta*	Acetoclastic pathway	−75.8	$CH_{3}COOH\to {CH}_{4}+{CO}_{2}$	[114]
Substrate: Methyl compound Environment: High salinity Bacteria: Methanocardiales, *Methanosphaera*	Methylotrophic pathway	−105.8	$CH_{3}OH+H_{2}\to {CH}_{4}+H_{2}O$	[115]
Substrate: Aceticlastic Environment: High temperature and low pH Bacteria: *Methanothermobacter*, *Thermactogenium*	Acetate oxidation	55.0	$CH_{3}COOH+2H_{2}O\to 4H_{2}+{2CO}_{2}$	[114]

**Table 8:** Effects of hydrocarbon substrate, electron acceptor, temperature, and other major factors on methanogenesis rate.

Condition	Variant	CH_4_ Production Rate	Refs.
Methane production rates in methanogenic-enrichment cultures originating from freshwater ditch sediments supplemented with different hydrocarbon substrates	Light oil	360–420 (nmol CH_4_/g TOC/day)	[125]
	n-alkanes (C_12_-C_18_)	350–560 (nmol CH_4_/g TOC/day)	[125]
	Heavy oil	170–250 (nmol CH_4_/g TOC/day)	[125]
	Paraffin (n-C_32_)	40–70 (nmol CH_4_/g TOC/day)	[125]
	BTEX (ethylbenzene, toluene)	30–70 (nmol CH_4_/g TOC/day)	[125]
	PAH (2-methylnapthalene)	20–50 (nmol CH_4_/g TOC/day)	[125]
Biogas upgrading via hydrogenotrophic methanogenesis in two-stage continuous stirred tank reactors at mesophilic and thermophilic conditions	35 °C, pH 7.74	66 ± 14 (mL/L day)	[126]
	55 °C, pH 7.82	247 ± 27 (mL/L day)	[126]
Microcosms were established by combining oil sands and formation water from the same oil sands reservoir as the only sources of microbial inoculum and organic carbon, amended with different electron acceptors	SO_4_^2−^	0.03 (μmol CH_4_/g oil sand/day)	[127]
	CO_3_^2−^	0.15 (μmol CH_4_/g oil sand/day)	[127]
	NO_3_^−^	-	[127]
Microbiological reduction of carbon dioxide into methane bioenergy through biochemical reaction by addition of zero-valent iron (ZVI) as an alternative electron donor in oil reservoir-production waters	ZVI	61.67 (μmol CH_4_/L oil reservoir production water/day)	[128]
	Without the addition of ZVI	0.60 (μmol CH_4_/L oil reservoir production water/day)	[128]

**Table 9:** Factors affecting the efficiency of crude oil gasification.

Impact Factor Categories	Factors	Influence
Environmental factor	Temperature, pH, Salinity	Microbial activity and metabolic pathways
	Trace elements, ammonia content, total nitrogen content, ammonia to alkalinity ratio, concentration of inhibitors like volatile fatty acids, ammonia, and heavy metals	Reaction rate of key enzymes
	Porosity, permeability, capillary force, and wettability	The contact area of the oil-water phase and microbial growth
Substrate factor	Carbon source	Metabolic pathways and methane production efficiency
	Electron acceptor	The continuation of the initial degradation and methanogenesis process
	Electron donor	Rate of reduction
Biological factor	Degradation enzymes such as alkyl succinate synthetase, phenyl methyl succinic synthetase	Degradation of alkanes, aromatic substituents
	Hydrogenases and dehydrogenases	Electron gain and loss
	Methyl-coenzyme M	Methane production efficiency

**Table 10:** Comparison of advantages and disadvantages of various machine-learning algorithms.

Common Machine Learning Algorithms	Advantage	Disadvantage	Refs.
Linear regression	High measurement rate and easy interpretation of results	Classification decision has an error rate	-
Logistic regression	The operation is simple and easy to understand and implement	Not suitable to deal with a large number of multi-class feature numbers or variables	-
Decision tree	Perform visual analysis, high running speed	Easy to overfit, easy to ignore the association within the data set	-
KNN	Easy to implement and high accuracy	High requirements on memory space	-
Artificial neural network	Strong parallel distributed processing capability and high accuracy	Black box process, hard to interpret the results	[154]
Random forest	Random forest can extend the importance of variables to all variables and identify these important variables, avoiding the elimination of important variables and high accuracy	Overfitting can occur in some noisy classification or regression problems	[155]

## Data Availability

Not applicable.
